# A Universal Formulary: Containing the Methods of Preparing and Administering Offiicinal and Other Medicines

**Published:** 1850-05

**Authors:** 


### REVIEWS.

Art. III.—*A Universal Formulary: containing the methods of preparing and administering officinal and other medicines. The whole adapted to physicians and pharmaceutists.* By R. Eglesfeld Griffith, M. D. Philadelphia: Lea & Blanchard. 1850.

This work is appropriately dedicated to Professors Wood and Bache, authors of the "Dispensatory of the United States of America." The labors of these gentlemen deserve all the respect and kindness that physicians can bestow on them, and Dr. Griffith has shown the possession of proper feeling, in this tribute to Drs. Wood & Bache.

We are not altogether confident that an increase of formularies is calculated to increase the armory of the physician, for the management of diseases. He is not the most useful in wealth, who has the most money; he that dispenses his means best, even if they are limited, is the best citizen. And that man's knowledge is most beneficial, that is used to the best advantage. Crowded formularies are apt to produce a jostling, and in the struggle to stand, it is possible that the worst may retain their feet. A formulary of mere recipes is not calculated to make thinkers; it does not materially aid the judgment, and may sometimes run away with the understanding.—The American practitioner has long been familiar with Ellis's Formulary, and it finds a place in almost every medical library, yet it would be difficult to say in what its special merits consist. Mayne's Dispensatory and Therapeutical Remembrancer," professed to contain "the entire lists of materia medica, preparations and compounds, with a full and distinct version of every practical formula, as authorized by the London, Edinburgh, and Dublin Royal College of Physicians in the latest editions of their several pharmacopœias," and the American edition, edited by the author of the "Universal Formulary," before us, was "revised, with the addition of the formulæ of the United States Pharmacopœia"? It seems to us that a work thus copious, did not stand much in need of a successor at present. The work of Mayne had a great many excellent features in it, and Dr. Griffith said it gave a clear and comprehensive view of all the officinal preparations directed by the authorities referred to." What created the demand for another work of this kind we do not know.

But though we are not convinced that this "Universal Formulary" was an absolute necessity, we are far from wishing to depreciate Dr. Griffith's work. He has bestowed a large amount of labor upon it, and every department of the book bears witness to the extended research and untiring industry of the author. That there is no ordinary share of useless lumber in the work is very certain, but there is also a great deal of matter that is very useful.

The formulary is preceded by an introduction, which "contains tables and observations on the weights and measures employed for pharmaceutical purposes in the United States and in foreign countries, and an explanation or vocabulary of the principal abbreviations and Latin terms used by physicians in writing prescriptions, followed by observations on the management of the sick room, with rules for the administration of the different classes of medicines."

"The formulary is arranged alphabetically, according to the pharmaceutic names adopted in the United States Pharmacopœia; but in each formula, the English appellations for the articles comprising it are used, and the quantities of these ingredients are expressed in words, and not in the usual pharmaceutic signs."

We consider the Introduction to the Formulæ one of the most valuable portions of this work. There is a reasonable portion of useful knowledge in it, compressed in a small space, and it is a kind of knowledge, in which practitioners are most likely to be defective. The remarks on the management of the rooms of the sick, though not an improvement upon Thomson's excellent work, are valuable to those who do not possess that work, and there are some useful suggestions on the proper administration of medicines.

Dr. Griffith says: "To facilitate a reference to the contents of the work, copious indices have been added, not only of the formulæ, but of the diseases for which they have been advised." Alas, the means for all the reference required by many, were too facile without the reflex indices! It is a railroad transit by the express train, and the locomotive will often fly from the track. For instance, under the name of ulcers, we have fifty-four distinct applications advised, when, if the experience and observation of the best surgeons of various epochs are to be relied on, not one of the fifty-four is useful or necessary.—Under the head of venereal ulcers, we have eight different applications, and among them is the following, purporting to be the recipe of an Italian physician, of no ordinary celebrity:

℞. Round brithwort,
Long brithwort,
Orris root, each half an ounce,
Opopanax,
Sagapenum, each two drachms,
Guiacum, four scruples,
Cloves, two drachms,
Camphor, three drachms,
Alcohol, ten ounces.

Macerate for twenty-four hours, and filter.

Our cotemporary, the Philadelphia Medical Examiner, "pleads to consummate ignorance in regard to the principles of combination, if any such there be, that suggested this formula," and we join in the plea. On what principle would a man employ this opopanax tincture? What condition of venereal ulcer would require its application? What are the principles of its action? We candidly acknowledge we know nothing on the subject. But we may take this very instance to show the evil tendencies of volumes of recipes. A practitioner wants a labor-saving machine to enable him to get along without thought. He takes Griffith's formulary, and is soon called upon to take charge of a venereal ulcer. Its peculiar condition, and the age, temperament, habits, mode of living, &c., of the patient, which he has to think of, are thrown aside, as unimportant matters; the important thing is that Griffith's formulary has eight applications for such ulcers, and the heedless, idle, unthinking routinist, in accordance with the instincts of such persons, hopes a great deal from each of them. He is very regular, and commences with the mercurial ointment. After trying that awhile, and fruitlessly, he goes to the next in the list—the lotion corrosive sublimate; then to the yellow wash; the ointment cyanide of mercury; ointment of the red iodide of mercury, black wash, compound tincture of opopanax, and winds up with a plaster biniodide potass. and opium, about which time, probably, the patient winds up the prescriber. A worse aid to the proper study of practical medicine, than a book of recipes, does not exist. Those who lean on such things for a staff, soon come to the want of crutches, and we might as reasonably expect high attainments, in the skilful treatment of disease, to show themselves in a man constantly in the habit of cutting "cures" from the columns of newspapers, as in one who consults a book of formulæ in order to aid his prescribing powers. This is not the proper method for the study of medicine; a fatal objection to it is found in the fact that it loses sight of the age, sex, temperament, habits, &c , of the patient, and leads men to prescribe for names of disease, instead of the symptoms. The practitioner, who is properly alive to his duties and responsibilities, will never content himself with practices of this kind; he will survey all that must be known of the case, in order to make up a judgment upon it, and physicians of this kind generally have knowledge enough of pharmacy and therapeutics, to prescribe, without resorting to the drilled matters of a formulary. The consequence is, that these books can do no great deal of good for those who could use them with judgment, but may do much harm in the hands of those who are unfit to prescribe either with or without the formulary. In the multitude of prescriptions for the same disease, some of which may be salutary in some circumstances, and baleful in others, a routinist may never strike the right one, except by the merest accident. We earnestly wish that some scientific Bailey could be found, of the capacity of the one appointed to stop the importation of adulterated drugs, who would superintend the "customs" of formularies, and restrain their contents within reasonable and salutary bounds.

But though we thus feel on the subject of formularies, we are not unmindful of certain merits in Dr. Griffith's work. We cordially join in the commendation of the "Medical Examiner," on the following points: "A large amount of useful and selected matters, well arranged, with the original observations of the author interlarded, is made to precede and follow the formulary proper:—among these are the subject of weights and measures, domestic and foreign; specific gravity; temperatures; vocabulary of words employed in prescriptions; observations on the management of the sick room; doses of medicines; rules for the administration of medicines; management of convalescence and relapses; dietetic preparations; list of incompatibles; posological table; officinal preparations and directions; bloodletting; poisons; index of diseases and their remedies, &c., &c."

The portions of Dr. Griffith's work, which treat of these subjects are really valuable, and are worthy of attention. They may be consulted with advantage by those who will feel no inclination to resort to the formulary, and if that part of the work were a sealed volume, the remainder of it may be considered a valuable contribution to practical medicine. It is very greatly in advance of all its competitors, and is worthy of confidence and use in the portions we have commended.

This work consists of 567 pages; it is well printed and substantially bound, and is quite free from the blemishes of typographical blunders. It may be purchased at the bookstore of Beckwith & Morton.

